# Left Ventricular Strains and Right Ventricular Longitudinal Shortening Are Associated in Healthy Adults—A Detailed Analysis from the Three-Dimensional Speckle-Tracking Echocardiographic MAGYAR-Healthy Study

**DOI:** 10.3390/life14111422

**Published:** 2024-11-04

**Authors:** Attila Nemes, Árpád Kormányos, Nóra Ambrus, Csaba Lengyel

**Affiliations:** Department of Medicine, Albert Szent-Györgyi Medical School, University of Szeged, Semmelweis Street 8, P.O. Box 427, H-6725 Szeged, Hungary; kormanyos.arpad@med.u-szeged.hu (Á.K.); ambrusnora@gmail.com (N.A.); lecs@in1st.szote.u-szeged.hu (C.L.)

**Keywords:** echocardiography, left ventricular, three-dimensional, speckle-tracking, strain, volume, healthy

## Abstract

Introduction: The right ventricle (RV) lies on the left ventricle (LV), and their shapes and movements are characteristic and significantly different. The aim of the present study was to investigate the relationship between three-dimensional speckle-tracking echocardiography (3DSTE)-derived LV strains, which represent LV contractility as quantitative features, and tricuspid annular plane systolic excursion (TAPSE) as determined by M-mode echocardiography, which represents the longitudinal movement of the RV, in healthy adults. Methods: A total of 79 healthy adults (mean age: 28.1 ± 6.3 years; 33 men) were enrolled in the present study. After two-dimensional Doppler echocardiography, 3DSTE-derived data acquisition was carried out in all cases, and detailed 3DSTE-based analysis was performed offline at a later date. Results: Reduced TAPSE was associated with increased global and basal LV radial strain (RS). Increased TAPSE was also associated not only with increased global and basal LV-RS but also with global LV longitudinal strain (LS). An increase in global LV-RS and global LV circumferential strain (CS) showed associations with other strains except for global LV-LS. An increase in global LV-LS did not show associations with other strains. Increased global LV-RS was associated with reduced TAPSE, while the degree of global LV-LS and global LV-CS did not show associations with TAPSE. Conclusions: Three-dimensional speckle-tracking echocardiography-derived LV-RS and LV-LS are associated with the longitudinal shortening of the RV represented by TAPSE in healthy adults.

## 1. Introduction

The heart and its cavities and valves form a complex unit and affect each other. The left (LV) and right ventricles (RV) can be considered the engines of systemic and pulmonary circulation; however, their morphology and consequent function differ considerably [1]. The LV is a heart chamber similar to a bullet, the segments of which contract and relax in all three directions (3Ds) of space during the heart cycle. The RV lies on the LV, and its shape and movement are characteristic and significantly different. While the LV is also supported by rotational mechanics, the RV is not, but it moves similarly to a bellows [2,3]. The basal part of the RV moves up and down longitudinally, similarly to the LV, represented by tricuspid/mitral annular plane systolic excursion (TAPSE/MAPSE) [2,3,4,5,6,7,8].

To understand the interaction between the LV and the RV in clinical settings better, even in healthy circumstances, the imaging studies currently available may help. Today, there are several imaging techniques available, including three-dimensional speckle-tracking echocardiography (3DSTE), that are suitable for detailed non-invasive functional examination, enabling physiological analysis [9,10,11,12]. LV contractility in 3D can be characterized by 3DSTE-derived specific LV strains. Moreover, the features of RV longitudinal shortening can be discerned according to M-mode echocardiography-derived TAPSE. The aim of the present study was to investigate the association between these parameters: 3DSTE-derived LV strains and TAPSE in healthy adults.

## 2. Subjects and Methods

Subjects: A total of 79 healthy adults (mean age: 28.1 ± 6.3 years; 33 men) were enrolled in the present study, who were recruited between 2011 and 2017. In all cases, a physical examination, a laboratory test, standard 12-lead electrocardiography (ECG), and two-dimensional (2D) Doppler echocardiography were performed with negative results. None of the participants were taking any medications, were smokers or obese, or had any known abnormalities or any medical conditions. After 2D Doppler echocardiography, 3DSTE-based data acquisition was carried out in all cases in accordance with the accepted practice, and a detailed 3DSTE-based analysis was carried out offline at a later date. The present retrospective study was part of the “Motion Analysis of the heart and Great vessels bY three-dimensionAl speckle-tRacking echocardiography in Healthy subjects” (MAGYAR-Healthy) Study. This study was organized to perform a physiological analysis between different, partly 3DSTE-derived variables in healthy circumstances (’Magyar’ means ’Hungarian’ in Hungarian language). This study was carried out in line with the Declaration of Helsinki (as revised in 2013), it was approved by the Institutional and Regional Biomedical Research Committee of the University of Szeged (number: 71/2011), and all healthy volunteers gave informed consent.

**Figure 1:**
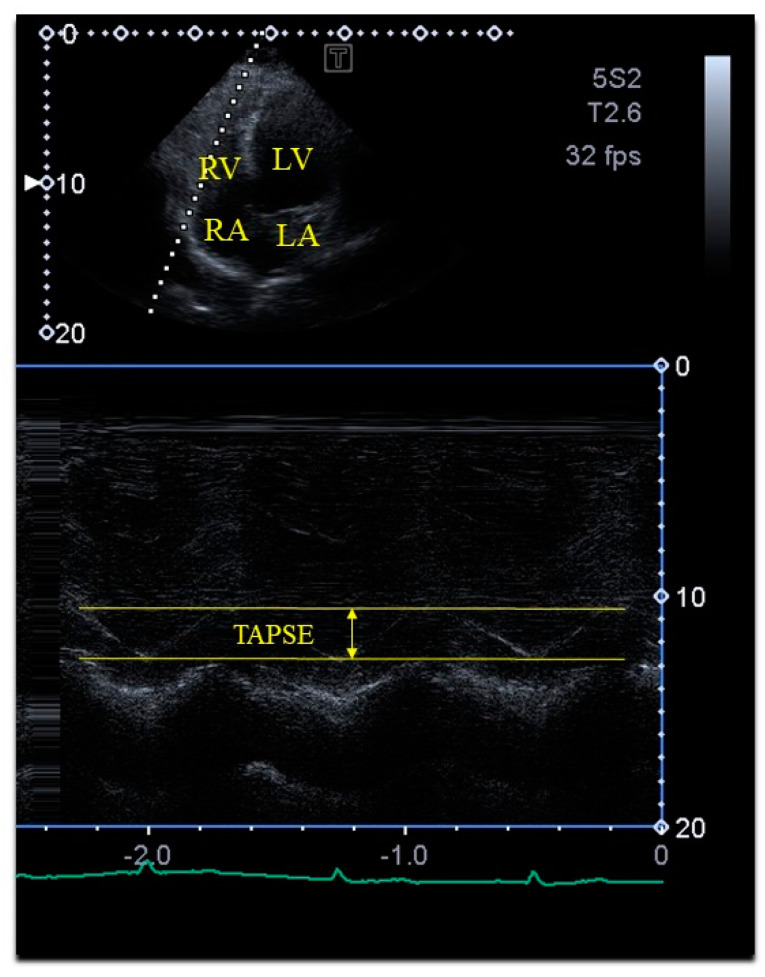
Measurement of tricuspid annular plane systolic excursion (TAPSE) by M-mode echocardiography in apical four-chamber view. Abbreviations: LA = left atrium; LV = left ventricle; RA = right atrium; RV = right ventricle; TAPSE = tricuspid annular plane systolic excursion.

Two-dimensional Doppler echocardiography: The 2D Doppler echocardiographic examinations were performed according to the latest professional guidelines. For all tests, a PST-30BT (1–5 MHz) phased-array transducer was attached to a Toshiba Artida^TM^ cardiac ultrasound device (Toshiba Medical Systems, Tokyo, Japan). All subjects to be tested were asked to lie in the left lateral decubitus position, after which the transducer was placed on their chest, and the examination was conducted from the typical parasternal and apical views. Significant valvular stenosis and regurgitation were ruled out using Doppler echocardiography. A pulsed Doppler was used to measure the transmitral flow E and A velocities and their ratio to evaluate diastolic function of the LV. Quantifications, including left atrial (LA) and LV measurements, and the LV ejection fraction (EF) were determined, the latter according to the Simpson method [2]. TAPSE was measured in the apical long-axis view as the movement of the lateral edge of the tricuspid annulus (TA) towards the RV apex in systole [3,4,5,6,7,8] (Figure 1).

**Figure 2:**
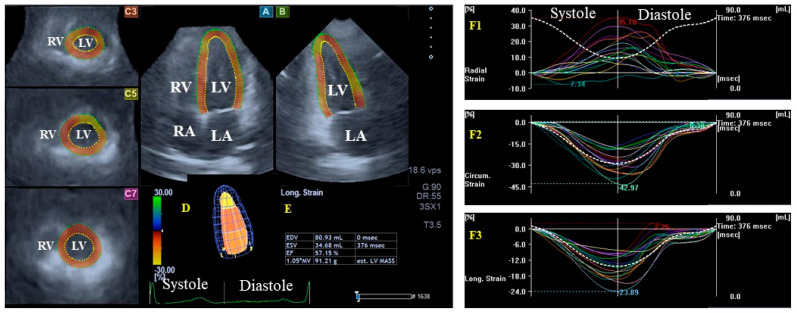
Assessment of the left ventricular (LV) strains by three-dimensional (3D) speckle-tracking echocardiography. Apical longitudinal four-chamber (A) and two-chamber (B) views and short-axis views at the basal (C3), midventricular (C5), and apical levels (C7) of the LV are presented together with a 3D cast of the LV (D) and the LV volumetric data calculated (E). Curves of time—global radial (F1), circumferential (F2), and longitudinal (F3) LV strains (colored lines) and time—change in the LV volume (dashed white line) during the cardiac cycle are shown together. Abbreviations: LA = left atrium; LV = left ventricle; RA = right atrium; RV = right ventricle; EDV = end-diastolic volume; ESV = end-systolic volume; EF = ejection fraction.

Three-dimensional speckle-tracking echocardiography: The 3DSTE studies were performed using the same Toshiba Artida^TM^ echocardiography device (Toshiba Medical Systems, Tokyo, Japan) attached to a PST-25SX matrix transducer. All cases were in sinus rhythm, and during the studies, the subjects lay in the left lateral decubitus position. Six 3D echocardiography subvolumes (datasets) were then acquired from the apical window to achieve the optimal image quality within six cardiac cycles, with healthy subjects restraining their breathing. After creating the auto-merged 3D full-volume dataset, data analysis was then performed using version 2.7 of 3D Wall Motion Tracking software (Ultra Extend, Toshiba Medical Systems, Tokyo, Japan) [9,10,11,12] (Figure 2).

Determination of LV strains: Using digitally acquired 3D echocardiographic databases, apical longitudinal four-chamber (AP4CH) and two-chamber (AP2CH) views and 3 cross-sectional views were used to determine the septal and lateral edges of the LV and the mitral annulus and the endocardial surface of the LV apex, and following sequential analysis and automatic contour detection, a 3D virtual model of the LV was created. Using this 3D LV cast, several global and basal regional unidirectional/unidimensional LV strains were determined to functionally characterize the whole LV [9,10,11,12]:

- Radial strain (RS), representing thickening/thinning of the LV;
- Circumferential strain (CS), representing narrowing/widening of the LV;
- Longitudinal strain (LS), representing shortening/lengthening of the LV.

Statistical analysis: All continuous variables were represented in mean ± standard deviation (SD) format, while categorical variables were presented in number/percentage format. Values were considered to be statistically significant when *p* < 0.05. To compare the groups, an analysis of variance (ANOVA) with Bonferroni correction and independent sample *t*-test s were performed. SPSS software version 22 (SPSS Inc., Chicago, IL, USA) was used for the statistical analyses.

## 3. Results

Clinical data: All of the clinical parameters were in the normal ranges for all healthy subjects. None of the subjects had borderline hypertension with LV hypertrophy (Table 1).

Two-dimensional Doppler echocardiography: All routine echocardiographic parameters were in the normal ranges. In no subject were wall abnormalities detected, and the wall motion score index proved to be 1. None of the subjects showed regurgitation greater than or equal to grade 1 or had significant stenosis in any valves.

Classification of subjects: The study group was classified according to their mean ± SD for TAPSE, global LV-RS (LV-GRS), global LV-CS (LV-GCS), and global LV-LS (LV-GLS). Accordingly, subgroups were created based on the lower (21 mm, 14.9%, 22.4%, and 13.6%, respectively) and upper (27 mm, 35.5%, 32.8%, and 18.6%, respectively) values of TAPSE, LV-GRS, LV-GCS, and LV-GLS.

Values for TAPSE and LV strains: Increased TAPSE was associated with elevated LV volumes. Reduced TAPSE was associated with increased global and basal LV-RS. Increased TAPSE was also associated not only with increased global and basal LV-RS but also with increased global LV-LS (Table 2).

Values for LV strains and TAPSE: An increase in global LV-RS and LV-CS showed associations with other strains except for global LV-LS. An increase in global LV-LS did not show associations with other strains. An increase in all strains was associated with an increased LV-EF, which was due to an increase in the LV-EDV in the case of global LV-RS; due to a reduction in the LV-ESV in the case of global LV-CS; and due to a non-significant reduction in both LV volumes in the case of global LV-LS. Increased global LV-RS was associated with reduced TAPSE, but the degree of global LV-LS and global LV-CS did not show any associations with TAPSE (Table 3).

## 4. Discussion

In recent decades, cardiovascular imaging has undergone significant development, with computer tomography and magnetic resonance imaging becoming widespread in addition to the previously dominant echocardiography. In addition, echocardiography has evolved, and new procedures such as speckle-tracking echocardiography and 3D echocardiography have emerged and spread. Three-dimensional speckle-tracking echocardiography, combining these two methods, is suitable for performing volumetric and functional strain-based measurements simultaneously using a digitally acquired database. This advantage makes it suitable for carrying out physiological examinations [9,10,11,12].

The LV consists of muscle fibers running perpendicular to each other subendocardially and subepicardially [13]. During systolic LV emptying, wall segments of the LV contract in the radial direction, shorten in the longitudinal direction, and narrow in the circumferential direction, represented by LV-RS, LV-LS, and LV-CS, respectively [14,15,16,17,18]. The great advantage of 3DSTE is that all LV strains can be calculated at the same time using the same acquired echocardiographic database [9,10,11,12]. Although 3DSTE is not yet sufficiently widespread, it is known and has been validated for the determination of LV strains [19,20], and 3DSTE-derived normal reference values have also been determined [21,22]. Associations between 3DSTE-derived LV strains and volumes and LV rotational mechanics and TAPSE have already been investigated within the frame of the MAGYAR-Healthy Study [23,24].

The deep muscle fibers in the walls of the RV are responsible for longitudinal movement from the base to the apex, which shortens the longitudinal axis of the RV and causes the TA (and the tricuspid valve itself) to move towards the apex, while the superficially located circumferential fibers, parallel to the TA, are responsible for movement towards the cavity of the RV (the “bellows-like” effect). These muscle fibers are connected to the superficial muscle fibers of the LV [4,5]. Although the muscular structure of the RV is different from that of the LV, it forms an integral unit with the TA, which has a special 3D saddle-shape structure. Not only does the TA behave like a sphincter but it is also longitudinally displaced, as represented by TAPSE, as well [25]. TAPSE is an established, easy-to-implement, M-mode-echocardiography-based, validated parameter that is suitable for characterizing the function of the RV and is considered a surrogate measure for RV strain as well. It is excellent for quantitative characterization of longitudinal shortening of the RV based on the movement of the basal part of the RV in the longitudinal direction [3,4,5,6,7,8].

Although the two chambers are interdependent, they differ in both their shape and structure, as well as in their function. It is important to understand what changes occur in the function of one chamber when that of the other changes. Accordingly, the relationship between 3DSTE-derived LV strains and M-mode echocardiography-derived TAPSE was examined in healthy adults. The main findings of the present study are that TAPSE findings both above and below the mean are associated with increased contractility of the LV, primarily in the basal region, in the radial direction (thickening/thinning), represented by LV-RS in healthy adults. Moreover, increased TAPSE is associated with elevated global LV-LS as well. These findings could suggest that a U-shaped curve for TAPSE demonstrates a compensatory increase in the radial contractility of the LV when longitudinal shortening of the RV is reduced (reduced TAPSE), provided that the dimensions and fractional area of the RV are not increased, suggesting an adaptive mechanism to balance the stroke volume in the right and left circulation. In cases of increased longitudinal shortening of the RV, both LV-RS and LV-LS proved to be elevated and were considered sub-phenomena of cardiac overwork. Moreover, the subjects with the highest global LV strains had higher LV-EFs, partly suggesting an effect of enhanced sympathetic stimulation. These results demonstrate the strong interaction between the two ventricles, even in healthy adults. However, further studies are needed to investigate the relationship between RV shortening and LV strains for different pathologies in which the left heart (such as in cases of myocardial ischemia, etc.) or the right heart is affected (such as congenital heart diseases, valvular heart diseases, cardiomyopathies, etc.).

Limitations: The following limitations arose:

- The image quality of 3DSTE is worse than that of 2D echocardiography, which may have affected our results.
- Although the rotational mechanics of the LV can be determined using the same LV cast, this was not considered the purpose of the present investigation. However, results on this from the MAGYAR-Healthy Study have already been published in detail. Moreover, the subject population used for this manuscript is partly the same [24].
- Although detailed analysis of other heart cavities can be performed in 3DSTE analysis, this study did not consider this to be its goal.
- As determining all LV strains by 3DSTE has been validated, this paper did not aim to do so.
- The body mass index of some subjects was above 25 kg/m^2^, meaning they were overweighted, which may have partly influenced our results. Although all the parameters tested in the present study were within the normal range, being overweight can have many effects, e.g., it can raise pulmonary artery pressure.
- Diastolic data on the LV or 3DSTE-derived parameters for the RV would enable an even more detailed analysis, which could be the topic of future investigations.

Conclusions. Three-dimensional speckle-tracking echocardiography-derived LV-RS and LV-LS are associated with longitudinal shortening of the RV, represented by TAPSE, in healthy adults. A compensatory increase in the radial contractility of the LV, as represented by LV-RS, could be detected where longitudinal shortening of the RV was reduced. Moreover, in cases of increased RV longitudinal shortening, not only the radial contractility of the LV, as represented by LV-RS, but also LV-LS, representing the longitudinal function of LV, proved to be elevated and were considered sub-phenomena of cardiac overwork.

## Figures and Tables

**Table 1:** Clinical and two-dimensional echocardiographic data.

Data	Measures
Clinical data	
*n*	79
Mean age (years)	28.1 ± 6.3
Males (%)	33 (42)
Systolic blood pressure (mmHg)	121.5 ± 3.6
Diastolic blood pressure (mmHg)	77.9 ± 2.7
Heart rate (1/s)	70.2 ± 2.0
Weight (kg)	73.1 ± 14.8
Height (cm)	168.6 ± 10.2
Body surface area (m^2^)	1.85 ± 0.35
Body mass index (kg/m^2^)	25.6 ± 1.8
Estimated pulmonary artery pressure (mmHg)	20.3 ± 2.8
Two-dimensional echocardiographic data	
LA diameter (mm)	36.9 ± 3.3
LV end-diastolic diameter (mm)	48.2 ± 3.6
LV end-systolic diameter (mm)	32.5 ± 3.4
LV end-diastolic volume (mL)	106.1 ± 23.8
LV end-systolic volume (mL)	38.5 ± 9.4
Interventricular septum (mm)	9.1 ± 1.2
LV posterior wall (mm)	9.2 ± 1.3
LV ejection fraction (%)	64.4 ± 4.2
Early diastolic mitral inflow velocity—E (cm/s)	82.9 ± 15.1
Late diastolic mitral inflow velocity—A (cm/s)	55.3 ± 10.4
Tricuspid annular plane systolic excursion (mm)	23.7 ± 2.9

Data are presented as numbers (percentages) or means ± standard deviation. LA = left atrium; LV = left ventricle.

**Table 2:** Tricuspid annular plane systolic excursion and left ventricular volumes and strains in different tricuspid annular plane systolic excursion groups.

	All Subjects (*n* = 79)	TAPSE ≤ 21 mm (*n* = 20)	21 mm < TAPSE < 27 mm (*n* = 44)	27 mm ≤ TAPSE (*n* = 15)
LV-EDV (mL)	85.7 ± 20.7	82.3 ± 23.8	82.8 ± 17.4	98.9 ± 19.8 *†
LV-ESV (mL)	36.2 ± 10.2	34.4 ± 12.2	35.0 ± 8.8	42.1 ± 8.5 *†
LV-EF (%)	57.9 ± 5.8	58.8 ± 6.6	57.8 ± 5.4	57.1 ± 5.5
LV mass (g)	164.1 ± 31.6	160.8 ± 28.1	164.3 ± 31.4	167.8 ± 35.7
global LV-RS (%)	25.2 ± 10.3	29.6 ± 13.0	22.7 ± 8.5 *	26.8 ± 8.6
basal LV-RS (%)	30.7 ± 13.1	35.3 ± 14.9	27.5 ± 12.1 *	34.2 ± 10.4 †
global LV-CS (%)	−27.6 ± 5.2	−27.8 ± 5.6	−27.7 ± 5.2	−27.1 ± 4.6
basal LV-CS (%)	−25.1 ± 4.7	−26.3 ± 5.3	−24.7 ± 4.5	−25.0 ± 4.4
global LV-LS (%)	−16.1 ± 2.5	−16.3 ± 2.9	−15.5 ± 2.4	−17.5 ± 1.7 †
basal LV-LS (%)	−20.7 ± 4.5	−21.8 ± 4.9	−20.4 ± 4.5	−19.9 ± 3.9
TAPSE (mm)	23.7 ± 2.9	20.3 ± 0.8	23.7 ± 1.3 *	28.5 ± 1.2 *†

* *p* < 0.05 vs. TAPSE ≤ 21 mm; † *p* < 0.05 vs. 21 mm < TAPSE < 27 mm. Abbreviations: LV = left ventricular; EDV = end-diastolic volume; ESV = end-systolic volume; EF = ejection fraction; RS = radial strain; CS = circumferential strain; LS = longitudinal strain; AS = area strain; 3DS = three-dimensional strain; TAPSE = tricuspid annular plane systolic excursion.

**Table 3:** Tricuspid annular plane systolic excursion and left ventricular strains in different left ventricular radial and circumferential strain groups.

	Global LV-RS ≤ 14.9% (*n* = 11)	14.9% < Global LV-RS < 35.5% (*n* = 56)	35.5% ≤ Global LV-RS (*n* = 12)	Global LV-CS ≤ −22.4% (*n* = 7)	−22.4% < Global LV-CS < 32.8% (*n* = 62)	−32.8% ≤ Global LV-CS (*n* = 10)	Global LV-LS ≤ −13.6% (*n* = 12)	−13.6% < Global LV-LS < −18.6% (*n* = 53)	−18.6% ≤ Global LV-LS (*n* = 14)
LV-EDV (mL)	71.2 ± 10.8	87.8 ± 20.2 *	89.4 ± 24.1 *	90.9 ± 18.5	85.1 ± 20.9	84.1 ± 20.4	90.9 ± 25.4	85.3 ± 1.95	82.9 ± 19.7
LV-ESV (mL)	33.2 ± 5.4	37.1 ± 9.9	34.9 ± 13.5	44.5 ± 9.0	36.6 ± 9.5 †	27.6 ± 8.8 †/††	41.3 ± 13.6	35.8 ± 8.6	33.4 ± 10.5
LV-EF (%)	53.2 ± 4.0	57.9 ± 4.9 *	62.1 ± 7.3 */**	50.7 ± 4.0	57.0 ± 3.6 †	68.1 ± 4.2 †/††	54.7 ± 5.4	57.8 ± 5.4	60.9 ± 5.6 ‡/‡‡
LV mass (g)	147.1 ± 30.7	167.1 ± 31.1	165.7 ± 29.3	169.0 ± 33.9	163.3 ± 31.6	165.0 ± 28.9	174.6 ± 29.3	165.2 ± 33.2	150.9 ± 20.9 ‡
global LV-RS (%)	10.6 ± 3.2	24.3 ± 5.0 *	43.1 ± 7.3 */**	18.7 ± 9.2	24.3 ± 8.5	34.2 ± 11.9 †/††	28.2 ± 13.0	24.4 ± 9.7	25.8 ± 9.6
global LV-CS (%)	−24.3 ± 4.9	−27.5 ± 4.5 *	−31.1 ± 6.2 */**	−19.0 ± 2.5	−27.0 ± 3.0 †	−26.5 ± 3.3 †/††	−26.1 ± 5.0	−27.3 ± 4.8	−29.8 ± 5.8
global LV-LS (%)	−15.5 ± 2.3	−16.2 ± 2.4	−15.9 ± 3.3	−15.7 ± 2.8	−16.0 ± 2.5	−17.2 ± 2.2	−12.5 ± 1.1	−15.9 ± 1.4 ‡	−20.0 ± 1.2 ‡/‡‡
TAPSE (mm)	23.8 ± 2.4	23.9 ± 2.9	22.2 ± 3.2 **	24.0 ± 2.0	23.8 ± 3.0	23.2 ± 3.0	22.8 ± 1.8	24.1 ± 2.9	22.8 ± 3.5

* *p* < 0.05 vs. global LV-RS ≤ 14.9%; ** *p* < 0.05 vs. 14.9% < global LV-RS < 35.5%; † *p* < 0.05 vs. global LV-CS ≤ −22.4%; †† *p* < 0.05 vs. −22.4% < global LV-CS < −32.8%; ‡ *p* < 0.05 vs. global LV-LS ≤ −13.6%; ‡‡ *p* < 0.05 vs. −13.6% < global LV-LS < −18.6%. Abbreviations: LV = left ventricular; EDV = end-diastolic volume; ESV = end-systolic volume; EF = ejection fraction; RS = radial strain; CS = circumferential strain; LS = longitudinal strain; AS = area strain; 3DS = three-dimensional strain; TAPSE = tricuspid annular plane systolic excursion.

## Data Availability

The data presented in this study are available on request from the corresponding author.
